# Reasons for academic cheating in a cohort of nursing students in Saudi Arabia: a cross-sectional study

**DOI:** 10.25122/jml-2023-0517

**Published:** 2024-04

**Authors:** Jazi Shaydied Alotaibi, Abdullah Alotaibi, Sharifa Alasiry, Bader Alrasheadi, Wdad Alanazy, Sameer Alkubati, Jordan Llego

**Affiliations:** 1 Department of Nursing, College of Applied Medical Sciences, Majmaah University, Al-Majmaah, Saudi Arabia; 2 PhD student in Nursing Science and Public Health, Department of Biomedicine and Prevention, Tor Vergata University of Rome, Rome, Italy; 3 Department of Medical Surgical Nursing, College of Nursing, University of Hail, Hail City, Saudi Arabia; 4 Department of Nursing, Faculty of Medicine and Health Sciences, Hodeida University, Hodeida, Yemen; 5 Nursing Department, College of Nursing and Midwifery, University of Luzon, Dagupan City, Philippines

**Keywords:** academic cheating, reasons, nursing, students, Saudi Arabia

## Abstract

This cross-sectional study investigated the reasons behind academic cheating in a cohort of nursing students in Saudi Arabia. The study involved 482 nursing students from two government universities in Riyadh. We used a newly developed self-reported questionnaire called the Reasons for Cheating Scale (RCS) to collect data. The highest-scoring reasons for academic cheating in the study population included the desire to obtain high grades, encouragement from friends to cheat, and the perception that exams were too difficult. Male students scored significantly higher than female students for reasons such as not understanding the course material, unclear test questions and instructions, pressure from families to excel, difficulty of the course material, and ignorance of effective study methods (*P* < 0.05). Age also had a role, as students aged 15–20 years had significantly higher scores for the item “Exams are too hard”, whereas those aged ≥25 years had higher scores for “Difficulty of the course material” (*P* < 0.05). Additionally, students in the preparatory year had significantly higher scores than those in other years for reasons such as difficult exams, unclear test questions and instructions, fear of failing, difficulty of the course material, and the desire to please their families (*P* < 0.05). Overall, the desire to obtain high grades emerged as the main reason for academic cheating in our cohort of nursing students in Saudi Arabia. The findings suggest that sociodemographic characteristics, including sex, age, and academic year, should be considered when addressing the issue of cheating among nursing students.

## INTRODUCTION

Nursing staff are guided by a strong set of ethical principles that are the foundation of their practice. They are responsible for upholding the highest standards of ethical conduct towards their colleagues, profession, and, most importantly, patients during their work [1]. Graduating nurses are expected to have the necessary attitudes, knowledge, and competencies to provide quality patient care [2,3]. Although nursing has been consistently ranked as the most honest profession [4], numerous studies have indicated that cheating is common among nursing students [5–8].

Recent studies have shown that cheating and related behaviors remain very common. Abusafia *et al*. found that 80% of 201 nursing students at a public university in Malaysia had engaged in academic dishonesty at least once [5]. Similarly, Lovrić and Žvanut found that about 91% of 446 Croatian nursing students admitted to engaging in dishonest behaviors on two or more occasions in the classroom [7]. What makes cheating among nursing students more dangerous is that it can also occur in clinical settings [6].

Another crucial factor why cheating among nursing students needs to be investigated is that if they cheat during their studies, they may cheat soon after becoming nursing staff. McCabe *et al*. stated that undergraduate studies are a critical period for developing ethical perception [9,10]. Students cheating during this critical period may see cheating as acceptable at work in the future. Cheating behaviors in clinical settings include manipulating clinical data documentation, potentially compromising the integrity of patient care and posing significant risks to patient safety [11].

Students who resort to cheating are likely to lack the necessary knowledge and skills required to practice safely in clinical settings because they may not be prepared to provide competent and effective care to patients, potentially leading to adverse outcomes [12]. Previous studies have found different reasons why nursing students cheat. For example, the influence of their peers and friends is one of the main reasons [8,13]. Through peer effects, cheating becomes acceptable behavior in school, as students reconsider cheating as ethically acceptable [14]. Theart and Smit reported that most of their respondents (71%) expressed that the fear of losing social standing among their peers would drive them to participate in cheating behaviors [15]. Altogether, peers significantly shape attitudes and behaviors, and their influence is recognized as one of the primary reasons why students cheat [10].

Academic staff and the educational environment are also crucial factors influencing academic cheating among nursing students [13,16]. Academic staff failing to act responsibly to prevent or minimize this behavior could contribute to an environment favoring dishonest behaviors among students [17]. Faculty members failing to take appropriate disciplinary actions for cheating incidents creates a perception that dishonesty goes unpunished, leading students to believe that cheating is acceptable [10].

Another main reason for cheating among nursing students is the high pressure they feel during their studies. Students work under high pressure to secure good jobs to satisfy their families, leading them to study under high pressure to achieve high grades [10]. Many other studies have also indicated the pressure to obtain a high grade point average as one of the main reasons why nursing students cheat [13,16,18]. Time pressure also has a significant contribution [11,13,18]. Students often face time constraints when preparing for examinations, leading to feelings of pressure and stress. In such cases, the perceived lack of time for adequate studying may drive some students to cheat as a shortcut to achieving their desired grades.

Several previous studies have examined the prevalence of cheating among nursing students. However, only a few have investigated their reasons for cheating. There is a significant gap in the literature regarding why nursing students cheat in Saudi Arabia. To address this research gap, this study aimed to identify the reasons for academic dishonesty among nursing students in Saudi Arabia and examine differences in the most common reasons by sex, and year of study. Addressing the reasons for cheating among nursing students is essential for maintaining the nursing profession’s integrity and ensuring that future nurses are well-prepared and ethical in their practice in Saudi Arabia. In addition, understanding academic cheating behaviors among Saudi nurses may help the country achieve academic integrity, which is essential for improving nursing education.

## METHODS

### Study design, sampling, and setting

This study used a cross-sectional design. Study participants were selected from two public universities in Ar-Riyadh, the largest region of Saudi Arabia. We used the Lynch formula to determine the sample size with a confidence interval of 95%, a margin of error of 5%. Given that the population size was 390 and 452 for university A and university B, respectively, the required number of respondents was at least 194 and 208, respectively. Between May and July 2022, we invited 842 nursing students from the two universities to participate in the survey. In total, 485 students agreed to participate, yielding a response rate of 57.60%. Three participants were removed because they did not complete the questionnaires. Therefore, 482 participants were included in this study. We included nursing students in their first (preparatory), second, third, and fourth years of a bachelor’s degree and excluded nursing students in their internship. We also excluded students who did not provide informed consent and those who did not finish the questionnaire.

To estimate the test–retest reliability, the first 50 participants completed the survey questionnaire package twice within 14 days. These participants were subsequently excluded from the study.

### Data collection

Data were collected online, using Google Surveys. After obtaining permission from the responsible authorities at the universities, a survey link was sent to eligible students’ email addresses, along with an introductory information sheet explaining the study’s purpose and procedure in plain language, and an agreement box indicating that they are giving their consent. Study participation was voluntary, and participants were assured of confidentiality, with no incentives or reprisals involved. They were informed of their right to withdraw from the study at any time and were provided with the contact details of the primary researcher for any questions or concerns. The collected data were handled carefully to maintain confidentiality, and the results were presented only in aggregate form without disclosing individual participants’ identities or personal information.

### Study tools

**Figure 1:**
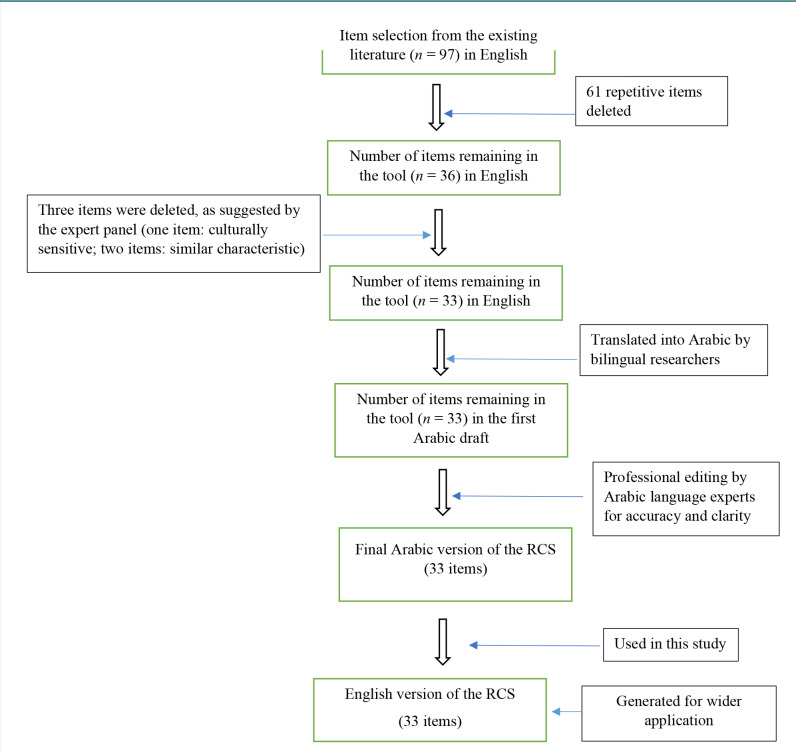
Flowchart of the development stages of the RCS

To examine nursing students’ reasons for cheating, we developed a new instrument, the Reasons for Cheating Scale (RCS), following the steps outlined in the proposal of Chalhoub-Deville (1996) to ensure its reliability and validity [19]. First, we determined a theoretical construct and item pool through an extensive review of previous studies [13,15,16,20] to identify items for the prospective questionnaire. The flow chart of the tool’s development is shown in Figure 1.

An initial set of 97 items was identified. After the removal of repetitive questions, 36 questions remained, which were sent to a panel of five external expert reviewers with extensive experience in nursing education in Saudi Arabia to determine the questionnaire’s validity. They reviewed the item pool and assessed each item for relevance, clarity, and alignment with the construct. Based on their suggestions, three questions were removed, and the wording of some of the questions was slightly revised. The final RCS questionnaire comprised 33 items (Table 1). All five experts with research backgrounds in psychometrics and nursing agreed on the test’s content validity. Moreover, they were asked to evaluate the 33 items on three separate Likert scales for essentiality, relevance, and clarity. Based on the Likert scale scores, the content validity ratio (CVR), content validity index for relevance (CVI-R), and content validity index for clarity (CVI-C) were determined. All 33 items of the RCS were found to have excellent content validity as indicated by a CVR of 0.9–1.0, a CVI-R of 0.9–1.0, and a CVI-C of 0.9–1.0.

**Table 1:** English version of RCS items

No.	Item
1	Lack of desire to study.
2	Students used to cheat in previous academic stages.
3	Not understanding the course material.
4	Exams are too hard.
5	Exams do not test what you have learned.
6	Some test questions and test instructions are unclear.
7	Weak punishment for cheating.
8	Not preparing well before exams.
9	Not paying attention in class, given that students know that they can eventually cheat when an exam comes.
10	Teacher’s lack of competence in explaining the subject material.
11	The existence of family circumstances hinders students from being adequately prepared for the exam.
12	Fear of failing the exam.
13	High pressures and expectations from students’ families to excel in their studies.
14	Having more than one exam on the same day.
15	Lecturers or invigilators leave the examination room during the exams.
16	The low educational level of students’ families.
17	Lack of awareness or guidance about the harmful effects of cheating and the importance of students being honest.
18	A large number of academic subjects that students study in a single semester.
19	Students who are friends feel obligated to help each other during exams.
20	Low self-confidence.
21	The difficulty of the course material.
22	Dissatisfaction with the examination process.
23	Convergence of seats in the examination hall.
24	Use of modern technologies, such as cell phones, for cheating.
25	Desire to obtain high grades.
26	Desire to please the family with success and superiority.
27	Encouragement of students’ friends to cheat in the examination.
28	Not having a suitable place to study at home.
29	Fear of bullying and reprimand from family and friends.
30	Cheating is morally acceptable among students.
31	Cheating is socially acceptable.
32	Indulgence of some lecturers while observing the exam.
33	Ignorance of the right way to study.

Then, a test–retest study with a 14-day interval was conducted to determine the RCS’s intraobserver reliability, involving 50 nursing students who were asked to complete the questionnaire and comment on its comprehensibility. The test–retest correlation value of the RCS was 0.96 (95% confidence interval (CI), 0.93–0.98), and its overall content validity index was 0.947.

Finally, the RCS was transferred into Arabic, and this version also underwent translation and back translation to develop an English version. We used the original Arabic version in the study. Internal consistency and homogeneity were tested using Cronbach’s alpha and item-level Cronbach’s alpha if item deleted (CAID). The internal consistency was excellent, as indicated by a Cronbach’s alpha of 0.961. Moreover, all 33 items of the RCS were statistically relevant and contributed almost identically to the overall reliability, as implied by very similar values of the CAID (range, 0.960–0.962).

### Data analysis

The data were analyzed using four software: SPSS v.23.0 (IBM Corp), JASP v.0.17.0.0 (University of Amsterdam), Factor v.12.03.02 (Rovira i Virgili University), and Jamovi v.2.3.18 (The Jamovi Project). We used descriptive statistics, such as the mean and s.d., and inferential statistics, such as the independent samples *t*-test and one-way analysis of variance (ANOVA). Assumptions were tested using Bartlett’s test of sphericity, the determinant, the Kaiser–Meyer–Olkin (KMO) criterion, Kaiser’s single-variable measure of sampling adequacy, and overall item analysis.

To interpret survey responses, the following measures were used for means: 1.00–1.80, totally disagree; 1.81–2.60, disagree; 2.61–3.40, neutral; 3.41–4.20, agree; 4.20–5.00, totally agree.

## RESULTS

### Participant characteristics

Most of the participating nursing students were aged 20–24 years (57.5%), men (57.9%), single (97.1%), and from university B (54.8%) (Table 2). Most participants (57.5%) were second- or third-year students (Table 2).

**Table 2:** Participant characteristics

Characteristic	*n*	%
**Age, years**		
<20	172	35.7%
20–24	277	57.5%
25–30	33	6.8%
**Sex**		
Male	279	57.9%
Female	203	42.1%
**University**		
University A	218	45.2%
University B	264	54.8%
**Marital status**		
Married	14	2.9%
Single	468	97.1%
**Year of study**		
Preparatory	80	16.6%
Second	184	38.2%
Third	93	19.3%
Fourth	125	25.9%

The primary reasons for academic dishonesty among the study population are presented in Table 3. These reasons were ranked based on their average significance to the students (mean) and the range of replies (s.d.). The factors that had the highest average scores, indicating the most significant influence on cheating behavior, were the aspiration for achieving excellent grades and the aspiration to impress family members with accomplishments and superiority. Both of these factors had an average score of 3.19. The presence of these components indicates a high level of drive towards academic success, whether driven by personal goals or expectations from one’s family. This is supported by a wide variety of student perspectives, with a s.d. of >1.20.

**Table 3:** The ten highest-scoring reasons for academic cheating in the study population

Item no.	Item	Mean ± s.d.
25	Desire to obtain high grades.	3.19 ± 1.26
26	Desire to please the family with success and superiority.	3.19 ± 1.24
4	Exams are too hard.	3.11 ± 1.02
12	Fear of failing the exam.	3.10 ± 1.17
13	High pressures and expectations from students’ families to excel in their studies.	3.08 ± 1.18
21	Difficulty of the course material.	3.02 ± 1.15
18	The large number of academic subjects that students study in a single semester.	3.00 ± 1.26
6	Some test questions and test instructions are unclear.	3.00 ± 1.15
3	Not understanding the course material.	2.96 ± 1.13
33	Ignorance of the right way to study.	2.95 ± 1.28

The factors that contributed significantly to the perception of exams being too difficult, fear of failure, and high pressure and expectations from the family to perform academically are especially noteworthy. These factors had mean scores ranging from 3.08 to 3.11, indicating that the pressure of high-stakes testing and external influences may be factors in the occurrence of dishonest conduct.

The complexity of the course material, the extensive range of subjects covered within a single semester, and the lack of clarity in test questions or instructions resulted in average scores of approximately 3.00, indicating important but significantly lesser concerns compared to the reasons indicated before.

Lastly, the lack of comprehension of the course material and the lack of knowledge about effective study methods had the lowest average scores of 2.96 and 2.95, respectively. However, they also showed a high level of variability (s.d. of >1.13), suggesting that these issues are significant, but there is a wider range of opinions on how they contribute to cheating behavior.

### Differences in the reasons for academic cheating by sex and age

We found significant differences between sexes in the mean scores of the ten highest-scoring reasons for academic cheating among the study population (Table 3). Male students had significantly higher scores than female students for all significant reasons for cheating, including items 3 (“Not understanding the course material”; *t* = −3.614; *P* < 0.05), 6 (“Some test questions and test instructions are unclear”; *t* = 2.588; *P* < 0.05), 13 (“High pressures and expectations from students’ families to excel in their studies”; *t* = 0.049; *P* < 0.05), 21 (“Difficulty of the course material”; *t* = 0.015; *P* < 0.05), and 33 (“Ignorance of the right way to study”; *t* = 0.000; *P* < 0.05).

We also found significant differences by age in the mean total scores of the ten highest-scoring reasons for academic cheating among the study population (Table 4). Students aged 15–20 years had significantly higher scores than the other age groups for item 4 (“Exams are too hard”; *F* = 4.854, ANOVA *P* < 0.05). In addition, students aged ≥25 had significantly higher scores than the other age groups for item 21 (“Difficulty of the course material”; *F* = 3.441, ANOVA *P* < 0.05), as shown in Table 5.

**Table 4:** Sex-related differences in the mean total scores of the ten highest-scoring reasons for academic cheating in the study population

Item no.	Male	Female	*t*	df	*P* value
3	2.69 ± 1.11	2.32 ± 1.13	3.614	480	<0.001^*^
4	2.21 ± 0.98	2.06 ± 1.06	1.589	480	0.113
6	2.62 ± 1.14	2.34 ± 1.14	2.588	480	0.010^*^
12	2.30 ± 1.21	2.13 ± 1.11	1.614	480	0.107
13	2.35 ± 1.19	2.14 ± 1.17	1.973	480	0.049^*^
18	2.59 ± 1.29	2.37 ± 1.21	1.897	480	0.058
21	2.59 ± 1.17	2.33 ± 1.11	2.435	480	0.015^*^
25	2.06 ± 1.26	2.15 ± 1.26	0.827	480	0.409
26	2.12 ± 1.23	2.15 ± 1.26	0.205	480	0.837
33	2.72 ± 1.32	2.30 ± 1.17	3.580	480	<0.001^*^

* Statistically significant. Data expressed as mean ± s.d.

**Table 5:** Age-related differences in the mean total scores of the ten highest-scoring reasons for academic cheating in the study population

Item no.	≤20 years	20–24 years	≥25 years	*F*	df	*P* value
3	1.01 ± 2.68	1.19 ± 2.48	1.17 ± 2.27	2.541	2	0.080
4	0.95 ± 2.34	1.06 ± 2.05	0.91 ± 2.03	4.854	2	0.008^*^
6	1.08 ± 2.58	1.18 ± 2.47	1.24 ± 2.36	0.766	2	0.466
12	1.14 ± 2.30	1.21 ± 2.19	1.08 ± 2.21	0.445	2	0.641
13	1.18 ± 2.28	1.19 ± 2.24	1.17 ± 2.39	0.269	2	0.764
18	1.24 ± 2.54	1.27 ± 2.44	1.28 ± 2.72	0.886	2	0.413
21	1.18 ± 2.63	1.14 ± 2.37	1.04 ± 2.69	3.441	2	0.033^*^
25	1.19 ± 2.06	1.33 ± 2.09	1.04 ± 2.30	0.472	2	0.624
26	1.20 ± 2.16	1.28 ± 2.11	1.13 ± 2.18	0.116	2	0.890
33	1.25 ± 2.58	1.30 ± 2.54	1.27 ± 2.42	0.215	2	0.806

* Statistically significant. Data expressed as mean ± s.d.

### Differences in the reasons for academic cheating by year of study

There were significant differences in the mean total scores of the ten highest-scoring reasons for academic cheating among the study population by year of study (Table 6). Students in the preparatory year had significantly higher scores than those in other years of study for items 4 (“Exams are too hard”; *F* = 3.620, ANOVA *P* < 0.05), 12 (“Fear of failing the exam”; *F* = 1.199, ANOVA *P* < 0.05), 21 (“Difficulty of the course material”; *F* = 2.657, ANOVA *P* < 0.05), and 26 (“Desire to please the family with success and superiority”; *F* = 2.952; ANOVA *P* < 0.05). In addition, students in their preparatory or fourth year had significantly higher scores than those in other years of study for item 6 (“Some test questions and test instructions are unclear”; *F* = 2.838, ANOVA *P* < 0.05).

**Table 6:** Differences in the mean total scores of the ten highest-scoring reasons for academic cheating in the study population

Item no.	Preparatory year	Second year	Third year	Fourth year	*F*	df	*P* value
3	2.60 ± 1.19	2.60 ± 1.08	2.26 ± 1.16	2.64 ± 1.12	2.291	3	0.078
4	2.37 ± 0.98	2.16 ± 1.03	1.88 ± 0.97	2.21 ± 1.03	3.620	3	0.013^*^
6	2.60 ± 1.05	2.46 ± 1.13	2.25 ± 1.25	2.69 ± 1.14	2.838	3	0.038^*^
12	2.38 ± 1.23	2.25 ± 1.19	2.05 ± 1.18	2.24 ± 1.11	1.199	3	0.309^*^
13	2.37 ± 1.30	2.17 ± 1.14	2.19 ± 1.22	2.38 ± 1.13	1.078	3	0.358
18	2.65 ± 1.37	2.48 ± 1.27	2.34 ± 1.19	2.54 ± 1.22	0.901	3	0.440
21	2.72 ± 1.21	2.48 ± 1.17	2.23 ± 1.08	2.52 ± 1.12	2.657	3	0.048^*^
25	2.23 ± 1.31	2.09 ± 1.28	1.75 ± 1.15	1.75 ± 1.24	3.622	3	0.013^*^
26	2.28 ± 1.24	2.14 ± 1.26	1.81 ± 1.15	2.28 ± 1.26	2.952	3	0.032^*^
33	2.61 ± 1.21	2.64 ± 1.26	2.34 ± 1.45	2.51 ± 1.19	1.252	3	0.290

* Statistically significant. Data expressed as mean ± s.d.

## DISCUSSION

This cross-sectional study aimed to identify the reasons for academic dishonesty among nursing students in Saudi Arabia and examine differences among the most common reasons by sex, age, and year of study. We found that the most common reason for cheating was the desire or pressure to obtain high grades, inconsistent with some previous studies [10,16,20]. However, Kiekkas *et al*. similarly reported that among 660 Greek nursing students, one of the main reasons for cheating was to achieve high grades [13]. High grades provide students a sense of achievement, as studies have shown that their grades in specific courses or examinations significantly determine their achievement level [21]. In addition, this could be attributed to the strict competition in the labor market. Students think high grades or academic credentials provide better job prospects [22].

Regarding the desire to please their family with success and superiority, in previous studies students reported that their parents appreciated achievement more than kindness and happiness [23]. Moreover, Nunes *et al*. claimed that some parents believe the primary objective of attending school is to achieve high academic performance [24]. Receiving poor marks might be perceived as a significant blow to parents’ self-esteem, and there is a prevailing belief that achieving high academic marks is a prerequisite for attaining an improved quality of life, adding pressure on students to engage in academic cheating.

Another contributing factor for the respondents engaging in cheating was the examination’s perceived difficulty, consistent with the results of Kiekkas *et al*. [13]. This finding is also supported by Siampani, who found that students’ propensity for cheating is heightened when faced with assessments with significant consequences or having reduced expectations of achieving success due to their perceived inadequacy or anxiety related to tests [25].

The fear of exam failure could be associated with a dispositional inclination to evade failure in settings that involve success. This inclination arises from the perception that the experience of humiliation and disgrace resulting from failure is too burdensome [26]. Other factors include students’ frequent experience of being emotionally and mentally burdened by concerns, accompanied by heightened physiological arousal and unfavorable emotions, in anticipation of impending academic due dates and examinations. This condition often hinders their ability to effectively study and actively participate in daily activities, explaining why they engage in academic cheating as a cognitive shortcut to expedite their learning process [27]. Moreover, this is closely related to the fifth highest-scoring reason: the high pressures and expectations from students’ families to excel in their studies [26]. Hosseinkhani *et al*. reported that family-derived stress was the most important source of stress [28]. Students who consistently strive to achieve the standards set by their families and perceive a persistent gap between their efforts and the desired outcome may resort to academic dishonesty in the hope that achieving high scores will elicit satisfaction from their families [29].

This study revealed a disparity in cheating behavior between male and female students, with male students showing a greater tendency to cheat than female students. This finding is consistent with those reported by Hadjar, who found a higher incidence of academic dishonesty among male university students than their female counterparts [30]. Moreover, Keikkas *et al*. and Krueger found that more male nursing students were reported for cheating than their female counterparts [6,13]. However, Park *et al*. showed that sex did not affect cheating among nursing students in South Korea [20]. In psychology, it has been shown that men often engage in cheating due to impulsive tendencies [31].

Moreover, the top three reasons for cheating among male students were “not understanding the course material,” “some test questions and test instructions are unclear,” and “high pressures and expectations from students’ families to excel in their studies.” These reasons could be attributed to their inclination towards lenient perspectives on academic dishonesty, given that men have a greater tolerance and confidence in engaging in cheating behaviors [30]. Furthermore, female students may be apprehensive about the potential detection of their involvement in academic dishonesty and the resulting social stigma. In contrast, male students appear to show a more carefree, careless, or audacious attitude toward cheating, showing a relative disregard for the potential consequences that may arise from it being detected [32].

The reasons for “not understanding the course material” and “some test questions and test instructions are unclear” might be collectively associated with cognition. Although research shows no difference in IQ between men and women, study habits could greatly affect cognition [33]. Establishing consistent study habits is crucial for learners to enhance their performance in the educational domain. Likewise, students show autonomous study behaviors characterized by regularity and dedication when they get encouragement and support from their parents, instructors, and classmates. Establishing consistent study habits among learners facilitates their attainment of aims and objectives. In essence, individuals who lack consistent study habits may encounter challenges in their academic pursuits and may, therefore, experience academic setbacks [34].

Regarding “high pressures and expectations from students’ families to excel in their studies,” parents’ influence may be a significant factor contributing to students’ engagement in academic dishonesty. Some students believe they may fulfill their parents’ high academic standards or gain their approval by achieving good grades by engaging in dishonest practices. Sometimes, parents may inadvertently overlook their children’s academic aptitudes, placing them under excessive pressure. Consequently, children may cheat to avoid disappointing their parents [35].

Regarding age differences in reasons for engaging in academic cheating, this study showed that younger students are more inclined to cheat when faced with difficult examinations, inconsistent with Kiekass *et al*. and Theart and Theart, who reported that nursing students’ age was not associated with cheating [13,15]. By contrast, older students were more likely to cheat when faced with challenging course content, consistent with Wang and Zhang, who reported a positive correlation between age and the likelihood of committing various forms of academic misconduct [36]. Moreover, Ossai *et al*. asserted that a negative correlation existed between students’ age and academic integrity [37].

Consistent with the aforementioned assertion, this study conducted a deeper investigation into the disparity between academic year and academic cheating, demonstrating a clear inclination among preparatory students to engage in cheating. This observation is consistent with Isakov *et al*., who examined the role of environmental variables on cheating behavior, finding a higher prevalence of academic dishonesty among first-year students than their senior counterparts in secondary education [38]. Nonetheless, this phenomenon will revert to its original state upon the individual’s transition to college. Moreover, studies have demonstrated that individuals who transition to college and embark on their academic path as first-year students show a greater propensity to engage in acts of academic dishonesty. This study’s findings also support the claim that preparatory university students have a limited understanding of academic misconduct, often tend to prioritize external motivations, and demonstrate a lack of correlation between their goals and receptiveness to intervention [39].

### Study limitations

Although our study was conducted at two modern governmental universities, both were in the Riyadh region of Saudi Arabia. Studies with greater geographical coverage may provide more generalizable results. In addition, our study was quantitative and cross-sectional, but qualitative and longitudinal studies may better describe cheating among nursing students.

## CONCLUSION AND RECOMMENDATIONS

The highest-scoring reason for academic cheating among our cohort of Saudi Arabian nursing students was the desire to obtain high grades. Nursing students’ sociodemographic characteristics can be important for cheating and should be considered when examining the causes of academic cheating. Nursing colleges and institutions must actively manage academic cheating causes, and strategies should be used to investigate other causes. In addition, nursing educators’ managers should be aware of and pay attention to the causes of cheating among nursing students to manage and eliminate this problem. Strategies should be developed and taught to nursing faculty instructors to address cheating in nursing students and prevent their cheating. Nursing faculties’ ethical committees should play an active role in investigating and preventing cheating among nursing students. Nursing students must be provided with detailed descriptions of the consequences and punishment for cheating by nursing faculties at the beginning of their study and followed up. Future qualitative studies are needed to better describe other causes of cheating.

## Data Availability

The dataset generated during and analyzed during the current study are available from the corresponding author upon reasonable request.
